# The association of findings on brain computed tomography with neurologic outcomes following extracorporeal cardiopulmonary resuscitation

**DOI:** 10.1186/s13054-017-1604-6

**Published:** 2017-01-25

**Authors:** Jeong-Am Ryu, Chi Ryang Chung, Yang Hyun Cho, Kiick Sung, Gee Young Suh, Taek Kyu Park, Young Bin Song, Joo-Yong Hahn, Jin-Ho Choi, Hyeon-Cheol Gwon, Seung-Hyuk Choi, Jeong Hoon Yang

**Affiliations:** 1 Department of Critical Care Medicine, Samsung Medical Center, Sungkyunkwan University School of Medicine, 81 Irwon-ro, Gangnam-gu, Seoul, Republic of Korea; 2 Department of Thoracic and Cardiovascular Surgery, Samsung Medical Center, Sungkyunkwan University School of Medicine, 81 Irwon-ro, Gangnam-gu, Seoul, Republic of Korea; 3 Division of Pulmonary and Critical Care Medicine, Department of Medicine, Samsung Medical Center, Sungkyunkwan University School of Medicine, 81 Irwon-ro, Gangnam-gu, Seoul, Korea; 4 Division of Cardiology, Department of Medicine, Samsung Medical Center, Sungkyunkwan University School of Medicine, 81 Irwon-ro, Gangnam-gu, Seoul, Republic of Korea

**Keywords:** Brain computed tomography, Cardiopulmonary resuscitation, Extracorporeal membrane oxygenation

## Abstract

**Background:**

Limited data are available on imaging predictors of neurological outcomes after extracorporeal cardiopulmonary resuscitation (ECPR). We investigated the association of initial brain computed tomography (CT) findings with neurological outcomes following ECPR.

**Methods:**

Between February 2005 and December 2015, a total of 42 patients who underwent brain CT scans within 48 h after ECPR were analyzed. Loss of the boundary between gray matter and white matter (LOB) or cortical sulcal effacement (SE), gray-to-white matter ratio (GWR), and optic nerve sheath diameter (ONSD) were measured on initial brain CT. The primary outcome was the Cerebral Performance Categories (CPC) scale at discharge.

**Results:**

Of the 42 adult ECPR patients, 23 (54.8%) patients survived to discharge and 19 (45.2%) patients had good neurological outcomes (CPC 1 and 2). The area under the curve (AUC) of GWR in the basal ganglia (GWR-BG) was 0.792 (95% confidence interval (CI), 0.639–0.901, *p* = 0.001). ONSD (AUC 0.745; 95% CI, 0.587 – 0.867, p = 0.007) was 5.57 (interquartile range (IQR) 5.14 – 5.98) mm in the good neurological outcome group versus 6.07 (IQR 5.71 – 6.64) mm in the poor outcome group. LOB or SE were more often detected in the poor neurological outcome group (AUC 0.817; 95% CI, 0.682–0.952, *p* <0.001). The predictive performance of poor neurological outcomes of a composite of GWR-BG, ONSD, and LOB/SE was significantly improved (AUC 0.904; 95% CI, 0.773–0.973) compared to when each brain CT marker was considered separately (GWR-BG, *p* = 0.048; ONSD, *p* = 0.026; LOB/SE, *p* = 0.028).

**Conclusions:**

GWR, ONSD, and LOB/SE on initial brain CT scans are associated with neurological prognosis in patients who underwent ECPR. The new risk prediction model, which uses a composite of GWR, ONCD, and LOB/SE, could provide better information on neurologic outcomes in patients underwent ECPR.

**Electronic supplementary material:**

The online version of this article (doi:10.1186/s13054-017-1604-6) contains supplementary material, which is available to authorized users.

## Background

Unconscious patients admitted to intensive care units (ICUs) after cardiac arrest are at a high risk of death, and development of neurologic deficits is common among those who survive [1]. In particular, neurological outcomes have been investigated more in patients with stable vital signs and reversible causes of arrest after a return of spontaneous circulation (ROSC). In the setting of conventional cardiopulmonary resuscitation (CPR), several predictors of neurological outcome have been reported such as physical examination of brainstem reflexes, various serum markers, and electrophysiologic study results [2–4]. Additionally, brain imaging may be helpful for predicting neurological outcomes after cardiac arrest [5–11]. Recently, extracorporeal cardiopulmonary resuscitation (ECPR) has been increasingly utilized to supply oxygenated blood and hemodynamic support in the absence of spontaneous cardiac circulation. Similar to studies on conventional CPR, there have been several studies on neurological prognosis in patients receiving ECPR [12–17]. However, there are limited data available on prognostic imaging markers of neurological outcomes after ECPR [18]. Brain computed tomography (CT) is simple, cost-effective, and easily implemented after ECPR [5]. Therefore, we investigated the association of initial brain CT findings with neurological outcomes following ECPR.

## Methods

### Study population

This was a retrospective, single-center, observational study of adult patients who underwent ECPR during hospitalization at the Samsung Medical Center between February 2005 and December 2015. This study was approved by the Institutional Review Board of the Samsung Medical Center. The requirement for informed consent was waived due to the retrospective nature of the study.

**Fig. 1:**
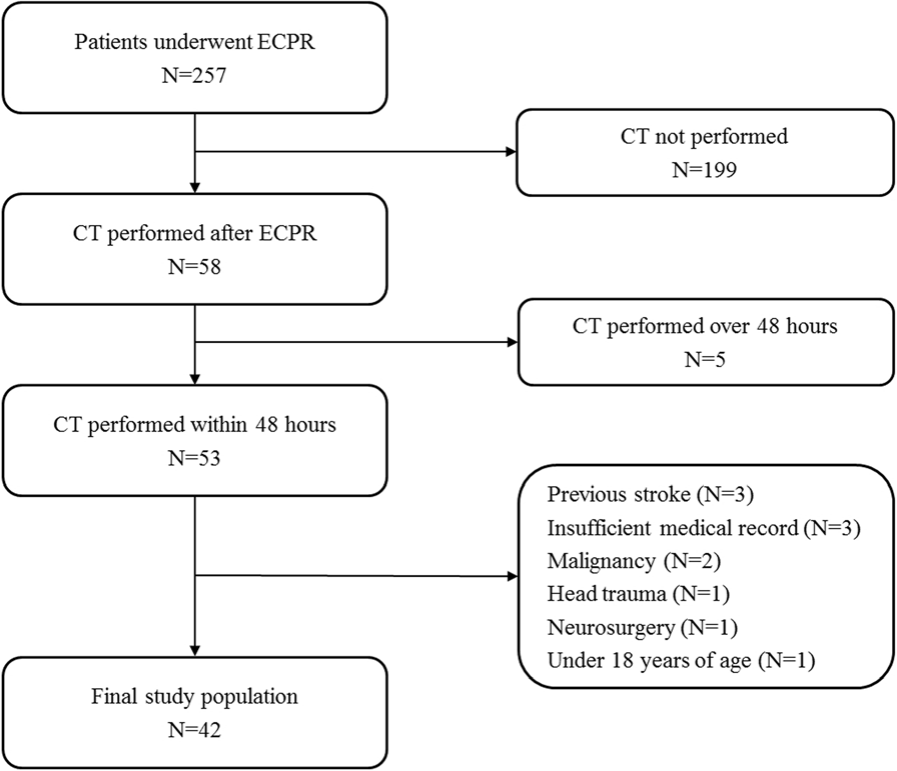
Study flow chart. *CT* computed tomography, *ECPR* extracorporeal cardiopulmonary resuscitation

We included patients who underwent ECPR during the study period, and who were unconscious (a score of <9 on the Glasgow Coma Scale on which scores range from 3 to 15, with lower scores indicating reduced levels of consciousness) on admission to the hospital after cardiac arrest and who were given a brain CT scan within 48 h after ECPR. Of these patients, we excluded patients under 18 years of age, patients with malignancy whose expected life span was less than 1 year, patients with insufficient medical records, and patients with a history of head trauma, neurosurgery, or a chronic neurological abnormality on ICU admission. A total of 257 patients underwent ECPR between February 2005 and December 2015. Of these, 53 patients underwent brain CT scans within 48 h after ECPR. Although a brain CT scan was performed after ECPR, a total of 11 patients were excluded from this study (Fig. 1). Finally, a total of 42 patients with cardiac arrest who were rescued by veno-arterial extracorporeal membrane oxygenation (ECMO) were analyzed in this study.

### Definitions and outcomes

**Fig. 2:**
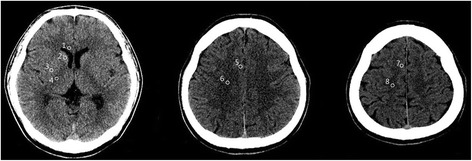
Brain computed tomography images showing measurements in Hounsfield units at the level of the basal ganglia (*left*), the centrum semiovale (*middle*), and the high convexity (*right*). The circular regions of interest are positioned in the genu of the corpus callosum (*1*), caudate nucleus (*2*), putamen (*3*), and posterior limb of the internal capsule (*4*) at the level of the basal ganglia and are positioned in the medial cortex (*5*, *7*) and medial white matter (*6*, *8*) at the centrum semiovale and high convexity levels, respectively

**Fig. 3:**
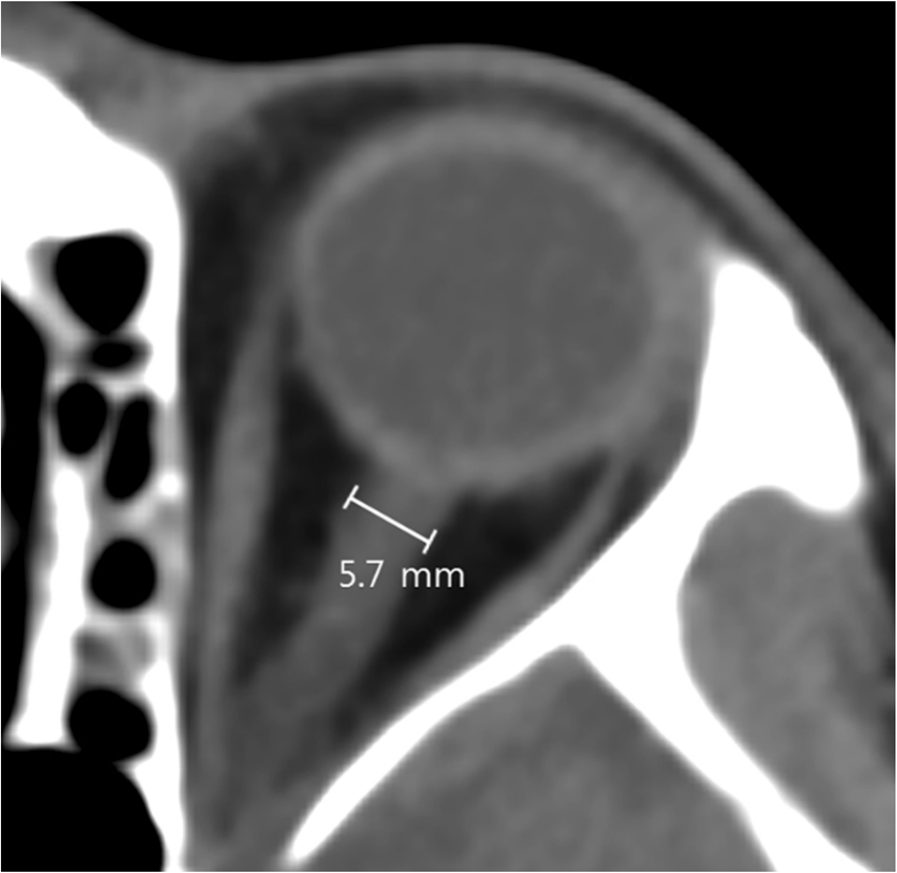
Measurement of optic nerve sheath diameter on initial brain computed tomography scan

ECPR was defined as both a successful veno-arterial ECMO implantation and pump-on with cardiac massage during the index procedure in patients with cardiac arrest in this study. Actually, when ROSC occurs during ECMO cannulation, the practitioner does not remove the already inserted cannula and does not stop the process of ECMO pump-on [14, 19]. ECMO pump-on was defined as the status that chest compression was stopped following successful ECMO implantation and activation. At this time, the ECMO flow was gradually increased until respiratory and hemodynamic statuses were stable. In this study, CPR duration was defined as the total time from onset to halting of chest compression. ROSC was defined for all rhythms as restoration of spontaneous rhythm that resulted in more than an occasional gasp, fleeting palpated pulse, or arterial waveform. CPR to ECMO pump-on time was defined as the time from initiation of chest compressions to the time at which the ECMO pump was turned on. Cardiac cause of arrest was presumed to be of cardiac origin unless it was known or likely to have been caused by trauma, submersion, hypothermia, drug overdose, asphyxia, exsanguination, or any other non-cardiac cause including intracranial hemorrhage, and terminal malignancy [20]. Acute coronary syndrome was diagnosed on the basis of electrocardiogram with typical symptoms, cardiac enzyme elevation, and coronary angiographic findings. The primary outcome was neurological status upon hospital discharge, as assessed with the Glasgow-Pittsburgh Cerebral Performance Categories (CPC) scale (1 to 5) [21]—CPC 1 and 2 were classified as good neurological outcomes, while CPC 3, 4, and 5 were considered poor neurological outcomes. We thoroughly reviewed medical records, and patients were graded on the CPC scale by two independent neurologists. Targeted temperature management was performed with surface cooling devices. We used a commercial temperature regulation system consisting of a hydrogel pad (Arctic Sun®; Medivance Corp, Louisville, CO, USA). Targeted temperature management was determined by intensivists in each ICU. Surface cooling and the degree of targeted temperature were determined by each intensivist in the ICU according to the SMC therapeutic hypothermia protocol [22]. If there were no particular problems, moderate therapeutic hypothermia (32–34 °C) was maintained. However, mild therapeutic hypothermia (35–36 °C) was performed if the patient had hemodyamic instability and bleeding complications during ECMO, or any complications after moderate therapeutic hypothermia. All of the recorded brain CT scans were taken within 48 h after ROSC in this study. After successful ECPR, a brain CT scan was not performed in patients who had a rapid recovery of mentality and neurological deficits. If not, a brain CT scan was performed to identify whether control of increased intracranial pressure was needed and to exclude intracranial hemorrhage before therapeutic hypothermia by an intensivist. A 64-channel scanner (LightSpeed VCT; GE Healthcare, Milwaukee, Wisconsin, USA) was used for all of the CT studies with a 5-mm slice width. Regions of interest (ROI) were identified by two independent neurologists. Investigators blinded to clinical information opened the CT scans for each patient using commercial image-viewing software (Centricity RA1000 PACS Viewer; GE Healthcare). The images were adjusted to the ‘brain’ window (window width 90 and window level 30) and comparable brain slices were identified at the level of the basal ganglia (BG) and two levels of the superior cortex, as described previously [5, 9, 10]. Circular regions of measurement (0.1–0.15 cm^2^) were placed over these ROI, and the average attenuation in Hounsfield units was recorded. At the BG level, the values were recorded bilaterally for the caudate nucleus (CN), putamen (PU), corpus callosum (CC), and posterior limb of the internal capsule (PIC) (Fig. 2). The gray-to-white matter ratio (GWR) in the BG (GWR-BG) was calculated according to a previously reported method as GWR-BG = (CN + PU) / (CC + PIC) [5, 9, 10]. We recorded values bilaterally for the medial cortex and medial white matter at the level of the centrum semiovale (MC1 and MWM1, respectively) and high convexity areas (MC2 and MWM2, respectively). The GWR in the cortex was calculated as GWR-CO = (MC1 + MC2) / (MWM1 + MWM2) [5, 9, 10]. Average GWR was calculated as the mean of the basal ganglia and cortical GWR (GWR-AV) = (CN + PU + MC1 + MC2) / (CC + PIC + MWM1 + MWM2) [5, 9, 10]. The GWR-BG was calculated using a simplified method as reported previously: GWR-simplified (SI) = PU / PIC [5, 8, 23]. The ROI were measured and averaged to yield the mean value, and the four GWRs (GWR-BG, GWR-CO, GWR-AV, and GWR-SI) were calculated as described above. Increasing cerebral edema indicates less attenuation of grey matter and a lower GWR. Optic nerve sheath diameter (ONSD) was measured at a distance of 3 mm behind the eyeball, immediately below the sclera in a perpendicular vector in reference to the linear axis of the nerve (Fig. 3) [11, 24, 25]. The images were changed to the ‘chest/abdomen’ window (window width 300 and window level 10) and were magnified threefold on the particular image slice which demonstrated the largest diameter of the optic nerve sheath [25]. ONSD was measured from one side of the optic nerve sheath to the other as a section through the center of the optic nerve [24]. The diameters measured for the patient’s left and right eyes were averaged to yield a mean value [24]. Loss of the boundary between gray matter and white matter (LOB) was evaluated at the level of the BG on the initial CT scans. The presence of cortical sulcal effacement (SE) was evaluated at the level of the centrum semiovale, and a positive SE sign was defined as SE detectable at this level [8, 23].

### Procedure

CPR was led by the CPR team of the hospital, and all facts related to the CPR scene were recorded by bedside nurses according to Utstein-style guidelines [26]. When CPR was performed for more than 10 min or in the event of unstable vital signs or recurrent cardiac arrest, the institutional rapid response team contacted the on-call ECMO team leader who, along with the CPR leader, assessed the patient and made a decision about whether to institute ECPR. ECPR was performed when a witnessed arrest was confirmed, the arrest persisted despite conventional CPR lasting for more than 10 min, and the event that caused the arrest was considered reversible [18]. Cases in which ECPR was deferred included a short life expectancy (<6 months), terminal malignancy, an unwitnessed collapse, limited physical activity, an unprotected airway, or CPR undertaken for more than 60 min at the time of initial contact. Age alone did not constitute a contraindication to ECPR [18].

The ECMO team consisted of cardiologists, cardiovascular surgeons, intensivists, special nurses, and perfusionists. Either the Capiox Emergency Bypass System (Terumo, Tokyo, Japan) or the Prolonged Life Support System (Maquet Cardiopulmonary, Hirrlingen, Germany) was used in all cases. A crystalloid solution such as normal saline or balanced solution was used for priming; no patient had blood-primed ECMO. A percutaneous vascular approach was tried initially in all cases using the Seldinger technique, but when percutaneous cannulation failed surgical cut-down exposure was performed [18]. The femoral vessels were the most common sites of vascular access, with the use of 14 to 17 French arterial cannulas and 20 to 24 French venous cannulas [19]. Cardiac compression was stopped once ECMO pump-on was successful during CPR. Anticoagulation was accomplished by a bolus injection of unfractionated heparin, followed by continuous intravenous heparin infusion to maintain an activated clotting time between 150 and 180 s. The initial number of revolutions per minute of the ECMO device was adjusted to achieve an ideal cardiac index greater than 2.2 L/min/m^2^ of body surface area, central mixed venous oxygen saturation above 70%, and a mean arterial pressure above 65 mm Hg [19]. Blood pressure was monitored continuously through an arterial catheter, and an artery in the right arm was used for arterial blood gas analysis to estimate cerebral oxygenation. After ECMO, the necessary steps were taken to treat the cause of the arrest, such as percutaneous coronary intervention, coronary artery bypass grafting, heart transplantation, non-coronary cardiopulmonary surgery, or non-cardiopulmonary surgery [19].

### Statistical analyses

All data are presented as medians and interquartile ranges (IQRs) for continuous variables and as numbers (percentages) for categorical variables. Data were compared using the Mann-Whitney *U* test for continuous variables, and the Chi-square test or Fisher’s exact test for categorical variables. The predictive performance of each brain CT marker was assessed using the area under the curve (AUC) of the receiver operating characteristic (ROC) curves of the sensitivity over 1 – specificity. AUCs were compared using the nonparametric approach published by DeLong et al. [27] for two correlated AUCs. The optimal cut-off values of each brain CT marker for predicting poor neurological outcome were obtained by ROC curve and Youden index [28, 29]. All tests were two-sided, and *p* values <0.05 were considered to indicate statistical significance. Data were analyzed using IBM SPSS statistics version 20 (IBM, Armonk, NY, USA).

## Results

### Baseline characteristics

The median patient age was 51 (range 25–82, IQR 34–65) years, and 29 patients (31.0%) were men. A cardiac cause of arrest was verified in 35 (83.3%) patients. Acute coronary syndrome was the main cause of cardiac arrest in 10 patients (23.8%) and 7 patients (16.7%) had a history of ischemic heart disease. Refractory arrhythmia was verified in 9 patients (21.4%). Thirty-five patients (83.3%) experienced cardiac arrest in the hospital and 7 patients (16.7%) had cardiac arrest in an out-of-hospital setting. The median CPR to ECMO pump-on time was 45 (IQR 27–55) min. Twenty patients (47.6%) had ROSC (median 7.5 min, range 1–47 min). Among these patients, only 4 patients had over 20 min ROSC duration and 13 patients had less than 10 min ROSC duration. Even 4 patients with ROSC >20 min had recurrent cardiac arrest before ECMO insertion, and no-flow time was sustained after the last ROSC. The baseline characteristics of ECPR patients are presented in Table 1 and the characteristics of cardiac arrest are given in Table 2. There were no significant differences between the two groups except for a history of diabetes mellitus at baseline and arrest-related characteristics.

**Table 1:** Baseline characteristics of comatose patients who underwent brain CT scans after ECPR

	Good neurological outcome (*n* = 19)	Poor neurological outcome (*n* = 23)	Total (*n* = 42)	*p* value
Age, years (median (IQR))	41.0 (32.0–70.5)	52.0 (43.0–60.5)	51.0 (34.0–65.0)	0.503
Gender, male (*n* (%))	14 (73.7)	15 (65.2)	29 (31.0)	0.739
Body mass index (kg/m^2^)	23.1 (21.1–25.7)	24.1 (22.2–28.5)	23.6 (21.5–26.7)	0.105
Medical history (*n* (%))				
Diabetes mellitus	1 (5.3)	10 (43.5)	11 (26.2)	0.005
Hypertension	3 (15.8)	11 (47.8)	14 (33.3)	0.048
Malignancy	3 (15.8)	5 (21.7)	8 (19.0)	0.709
Smoking	7 (36.8)	11 (47.8)	18 (42.9)	0.474
Previous myocardial infarction	0 (0)	2 (8.7)	2 (4.8)	0.492
Initial Glasgow Coma Scale (median (IQR))	3 (3–6)	3 (3–4)	3 (3–5)	0.518
Target temperature manage by surface cooling device (*n* (%))	7 (36.8)	9 (39.1)	16 (38.1)	0.879
Interval between ECPR and CT scan (*n* (%))				0.001
0–6 h	8 (42.1)	11 (47.8)	19 (45.2)	
6–12 h	0 (0)	2 (8.7)	2 (4.8)	
12–24 h	10 (52.6)	1 (4.3)	11 (26.2)	
24–48 h	1 (5.3)	9 (39.1)	10 (23.8)	
Laboratory data on admission (median (IQR))				
Initial lactate (mg/dl)	83.8 (51.4–127.0)	105.4 (85.6–136.9)	97.3 (68.5–132.4)	0.133
(mmol/l)	9.3 (5.7–14.1)	11.7 (9.5–15.2)	10.8 (7.6–14.7)	
Hemoglobin before ECPR (g/dl)	13.9 (10.5–15.6)	12.0 (11.0–14.0)	12.6 (10.9–14.6)	0.263
(mmol/l)	8.6 (6.5–9.7)	7.4 (6.8–8.7)	7.8 (6.8–9.1)	
Hemoglobin after ECPR (g/dl)	10.5 (8.8–11.9)	9.9 (8.8–11.1)	10.2 (8.8–11.5)	0.597
(mmol/l)	6.5 (5.5–7.4)	6.1 (5.5–6.9)	6.3 (5.5–7.1)	
Total bilirubin (mg/dl)	0.7 (0.5–1.2)	1.0 (0.5–1.2)	0.8 (0.5–1.2)	0.744
(μmol/l)	12.0 (8.6–20.5)	17.1 (8.6–20.5)	13.7 (8.6–20.5)	
Blood urea nitrogen (mg/dl)	16.7 (14.7–23.0)	17.2 (13.6–21.8)	16.7 (14.0–22.4)	0.745
(mmol/l)	6.0 (5.2–8.2)	6.1 (4.9–7.8)	6.0 (5.0–8.0)	
Creatinine (mg/dl)	1.2 (1.0–2.0)	1.3 (1.1–1.7)	1.3 (1.1–1.8)	0.533
(μmol/l)	106.1 (88.4–176.8)	114.9 (97.2–150.3)	114.9 (97.2–159.1)	

*CT* computed tomography, *ECPR* extracorporeal cardiopulmonary resuscitation, *IQR* interquartile range

**Table 2:** Characteristics of cardiac arrest in comatose patients who underwent brain CT scans after ECPR

	Good neurological outcome (*n* = 19)	Poor neurological outcome (*n* = 23)	Total (*n* = 42)	*p* value
Type of cardiac arrest (*n* (%))				0.999
Out-of-hospital cardiac arrest	3 (15.8)	4 (17.4)	7 (16.7)	
In-hospital cardiac arrest	16 (84.2)	19 (82.6)	35 (83.3)	
Bystander-witnessed cardiac arrest (*n* (%))	19 (100.0)	23 (100.0)	42 (100.0)	
Bystander-performed CPR (*n* (%))	17 (89.5)	23 (100.0)	40 (95.2)	0.199
First monitored rhythm (*n* (%))				0.714
Asystole	2 (10.5)	3 (13.0)	5 (11.9)	
Pulseless electrical activity	8 (42.1)	11 (47.8)	19 (45.2)	
Shockable rhythm (VT or VF)	8 (42.1)	9 (39.1)	17 (40.5)	
Defibrillation (*n* (%))	10 (55.6)	13 (56.5)	23 (56.1)	0.951
ROSC before ECMO insertion (*n* (%))	8 (44.4)	12 (52.2)	20 (48.8)	0.623
CPR to ECMO pump-on time, min (median (IQR))	43.5 (19.0–55.0)	47.0 (33.5–56.0)	45.0 (27.0–55.0)	0.430
Location of ECMO insertion (*n* (%))				0.507
Intensive care unit	6 (31.6)	5 (21.7)	11 (26.2)	
Cath room	3 (15.8)	7 (30.4)	10 (23.8)	
Emergency room	10 (52.6)	10 (43.5)	20 (47.6)	
Operation room	0 (0)	1 (4.3)	1 (2.4)	
Cardiac cause of arrest (*n* (%))	17 (89.5)	18 (78.3)	35 (83.3)	0.371
Acute coronary syndrome	3 (15.8)	7 (30.4)	10 (23.8)	
STEMI	1 (5.3)	3 (13.0)	4 (9.5)	
NSTEMI	1 (5.3)	3 (13.0)	4 (9.5)	
Unstable angina	1 (5.3)	1 (4.3)	2 (4.8)	
Cardiomyopathy	1 (5.3)	0 (0)	1 (2.4)	
Acute aortic syndrome	0 (0)	2 (8.7)	2 (4.8)	
Pulmonary thromboembolism	3 (17.6)	3 (13.0)	6 (14.3)	
Refractory arrhythmia	5 (26.3)	4 (17.4)	9 (21.4)	
Left ventricular wall rupture	4 (21.1)	1 (4.3)	5 (11.9)	
Other	1 (5.3)	1 (4.3)	2 (4.8)	

*CPR* cardiopulmonary resuscitation, *CT* computed tomography, *ECMO* extracorporeal membrane oxygenation, *ECPR* extracorporeal cardiopulmonary resuscitation, *IQR* interquartile range, *NSTEMI* non-ST segment elevation myocardial infarction, *ROSC* return of spontaneous circulation, *STEMI* ST segment elevation myocardial infarction, *VT* ventricular tachycardia, *VF* ventricular fibrillation

### Clinical and neurological outcomes

Among 42 adult cardiac arrest patients who underwent ECPR, successful ECMO weaning was achieved in 29 patients (69.0%) and survival to discharge was identified in 23 patients (54.8%). Of these 23 survivors, 19 patients (45.2%) had good neurological outcomes (CPC 1 and 2). The ICU mortality rate was 40.5% (17 patients). Of the 42 patients, target temperature management was performed in 16 patients (38.1%) using surface cooling devices.

### Brain CT findings

**Fig. 4:**
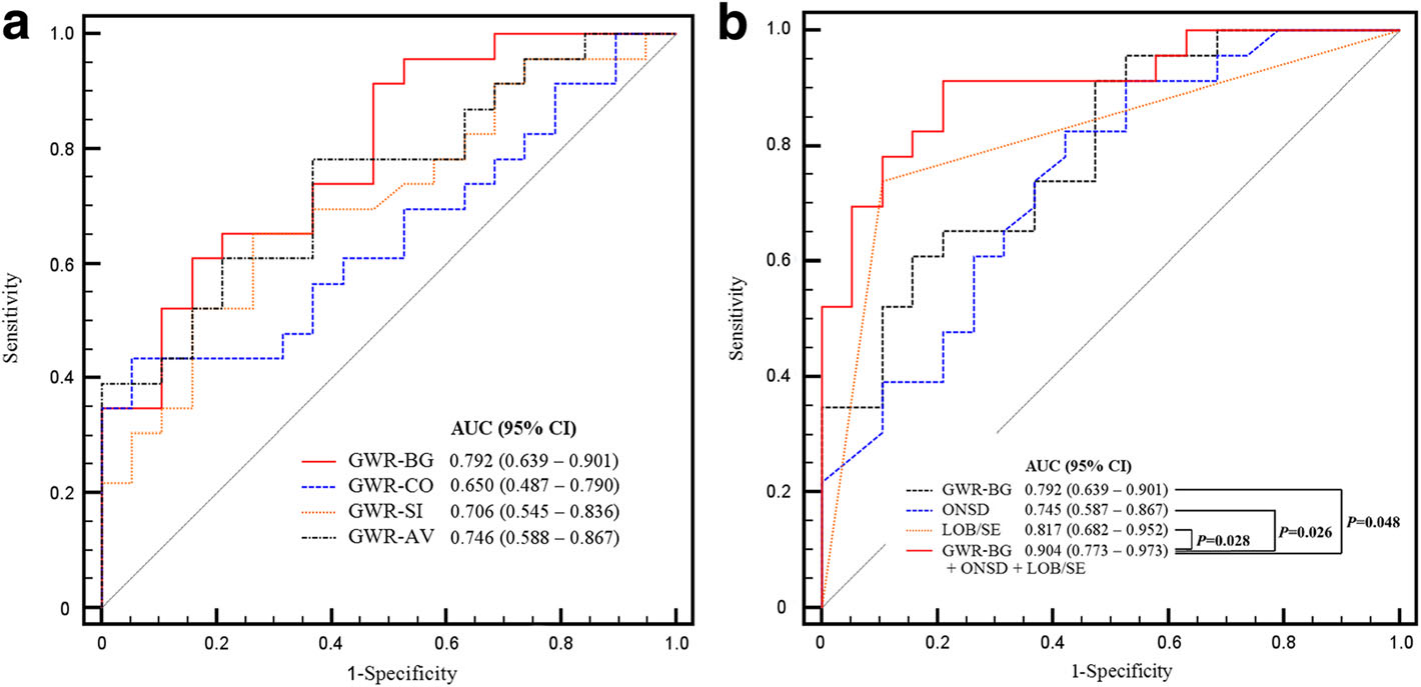
ROC curves for the prediction of poor outcomes. **a** ROC curves for the prediction of poor outcomes using the gray-to-white matter ratio of the basal ganglia (*GWR-BG*), cortex (*GWR-CO*), simplified (*GWR-SI*), and average (*GWR-AV*). **b** ROC curves for the prediction of poor outcomes using the GWR-BG, optic nerve sheath diameter (*ONSD*), loss of boundary between gray matter and white matter and cortical sulcal effacement (*LOB/SE*), and a combination of the three markers. *AUC* area under the curve, *CI* confidence interval

Although there were no significant differences between the average attenuations in Hounsfield units of each ROI, GWRs differed significantly between the two groups except for GWR-CO. GWRs were higher in the good neurological outcome group (Table 3). The ONSD was 5.57 (IQR 5.14–5.98) mm in the good outcome group versus 6.07 (IQR 5.71–6.64) mm in the poor outcome group (*p* = 0.007). LOB sign and SE sign were more likely to be detected in the poor neurological outcome group than in the good neurological outcome group (both *p* < 0.001). These CT markers of each outcome are shown in Table 3. The cut-off value, specificity, and sensitivity of these CT markers for poor outcome are shown in Additional file 1 (Table S1). For the ROC curve analysis for the prediction of poor neurological outcome, GWR-BG had the largest AUC (0.792; 95% confidence interval (CI), 0.639–0.901) among all the GWRs (Fig. 4a). Although there were no differences between the AUCs of GWR-BG, ONSD, and LOB/SE, the performance of a composite of these markers (AUC 0.904, Brier score 0.123, Goodness-of-fit (Hosmer-Lemeshow) χ^2^ = 7.562, *p* = 0.477) was more associated with poor neurological outcomes than the use of each brain CT marker alone (Fig. 4b). Eight patients (19.0%) had all three poor markers when poor markers of GWR-BG and ONSD were defined according to cut-off values in Additional file 1 (Table S1) (GWR-BG <1.23 and ONSD >5.86 mm, respectively). All of them had poor neurological outcome (sensitivity 34.8% and specificity 100%). The number of patients with two or more poor markers was 17 (40.5%). Of these patients, only one patient had good neurological outcome (sensitivity 69.6% and specificity 94.7%).

**Table 3:** Comparisons of the attenuation in regions of interest and of the gray-to-white matter ratio (GWR), optic nerve sheath diameter, and early computed tomography signs between groups

			Good neurological outcome (*n* = 19)	Poor neurological outcome (*n* = 23)	*p* value
Density of regions of interest, HU (median (IQR))					
Basal ganglia	Gray matter	CN	34.90 (32.25–38.10)	32.40 (28.20–35.60)	0.071
		PU	35.70 (33.40–38.05)	33.90 (31.25–35.9)	0.129
	White matter	CC	27.90 (24.20–30.60)	27.40 (25.55–29.75)	0.752
		PIC	27.70 (25.05–29.20)	27.30 (26.15–28.80)	0.950
Centrum semiovale (cortical)	Gray matter	MC1	31.90 (28.75–34.30)	30.80 (27.65–32.45)	0.306
	White matter	MWM1	24.9 (22.25–29.55)	25.30 (23.40–28.10)	0.667
High convexity area (cortical)	Gray matter	MC2	30.70 (28.85–33.80)	29.70 (28.25–32.05)	0.596
	White matter	MWM2	24.60 (21.85–27.05)	24.70 (22.00–27.45)	0.527
GWR (median (IQR))					
GWR-BG (basal ganglia)			1.31 (1.25–1.37)	1.21 (1.11–1.28)	0.001
GWR-CO (cortical)			1.23 (1.17–1.32)	1.20 (1.09–1.29)	0.098
GWR-SI (simplified)			1.29 (1.25–1.38)	1.21 (1.13–1.29)	0.023
GWR-AV (average)			1.29 (1.23–1.31)	1.21 (1.10–1.26)	0.007
Optic nerve sheath diameter, mm (median (IQR))			5.57 (5.14–5.98)	6.07 (5.71–6.64)	0.007
LOB at level of basal ganglia (*n* (%))			2 (10.5)	15 (65.2)	<0.001
SE at level of centrum semiovale (*n* (%))			2 (10.5)	15 (65.2)	<0.001

*CC* corpus callosum, *CN* caudate nucleus, *HU* Hounsfield units, *IQR* interquartile range, *LOB* loss of boundary between gray matter and white matter, *MC* medial cortex, *MWM* medial white matter, *PIC* posterior limb of the internal capsule, *PU* putamen, *SE* cortical sulcal effacement

## Discussion

ECPR has been increasingly utilized to supply oxygenated blood and hemodynamic support in the absence of spontaneous cardiac circulation. Therefore, ECPR may be considered in settings where it can be rapidly implemented among selected cardiac arrest patients who have not responded to initial conventional CPR [30]. In addition, ECPR had a short-term and long-term survival benefit over conventional CPR in patients with in-hospital cardiac arrest of cardiac origin [14, 31]. Similar to studies on conventional CPR, there have been several studies on neurological prognosis in patients receiving ECPR [12–17]. However, limited data are available on prognostic imaging markers of neurological outcomes after ECPR [18].

In the present study, we evaluated whether GWR, ONSD, and LOB/SE as measurable variables on initial brain CT scans were associated with neurological outcome in patients who underwent ECPR. Although there were no significant differences between the average attenuation in Hounsfield units of each ROI, GWRs (except GWR-CO) on initial brain CT were higher in the good neurological outcome group than in the poor outcome group. ONSD on initial brain CT was correlated closely with neurologic outcome and was greater in the poor neurological outcome group. LOB sign and SE sign were more likely to be detected in the poor neurological outcome group than in the good neurological outcome group. GWR, ONSD, and LOB/SE were associated with neurological outcome after ECPR, and these three CT markers had statistically similar predictive power on poor neurological outcome. Furthermore, a composite of GWR-BG, ONSD, and LOB/SE was a significantly greater predictor of poor neurological outcomes than were each brain CT marker alone.

There have been reports on several predictors of neurological outcome after conventional CPR [2]. Absence of brainstem reflex and motor response other than extensor posturing over a period of 72 h and myoclonic status epilepticus are markers of poor outcomes after cardiac arrest [2, 3]. Elevated serum S-100B and neuron-specific enolase levels and bilaterally absent somatosensory evoked potentials appear to have prognostic value in predicting a poor outcome [2, 3]. In addition, there have been reports of neuroimaging studies such as brain CT, brain magnetic resonance imaging, and magnetic resonance spectroscopy after conventional CPR [2]. In particular, brain CT scans may be helpful for predicting neurological outcomes after ECPR because brain magnetic resonance imaging is an expensive and time-consuming procedure and cannot be immediately performed because of ECMO equipment in patients receiving ECPR [5]. Sedation may confound outcome prediction in survivors of cardiac arrest [32]. Sedative medications are commonly used in comatose CPR survivors for 72 h and are an important confounder [32]. Some clinical signs and examinations such as a motor response to noxious stimuli, corneal reflex, caloric testing, and electroencephalography may also be confounded by sedation [2, 32]. Therefore, brain CT may be more helpful for predicting neurological outcome in ECPR patients under sedation. However, there are limited data on prognostic markers of brain CT for neurological outcomes after ECPR [13, 18].

Some signs on brain CT have been associated with ischemic cerebral insult and poor neurological outcome [5, 7–11]. The LOB and SE signs are associated with ischemic brain damage and poor outcome after cardiac arrest [8]. These signs were also associated with poor neurological outcomes after ECPR in this study. However, identification of these signs may be subject to the abilities of each investigator, and these signs are not quantifiable. GWR was associated with neurological outcome in patients receiving both conventional CPR and ECPR [5, 7–11]. GWR may be explained by the quantitative association of cerebral edema following hypoxic injury with increased intracranial pressure. Similar to other studies, GWRs were significantly different and the attenuation in Hounsfield units in the ROIs were not statistically different between the two groups in this study [5, 9, 10]. In previous studies, average GWR was a better predictor of neurologic outcome than the GWRs of single regions such as BG or the cerebellum alone [5, 9–11]. However, GWR-BG had the largest AUC for prediction of poor neurological outcome among the GWRs in this study. The predictive power of GWR-AV may have been decreased because GWR-CO had little association with poor neurological outcomes in this study. GWRs may be a useful predictor of neurological outcome after ECPR, although the GWR cut-off values were different in different studies [5, 9, 10].

Measurement of ONSD has been proposed as an alternative method for the detection of increased intracranial pressure [11]. The optic nerve is surrounded by cerebrospinal fluid because it is a part of the central nervous system. Therefore, increased intracranial pressure will be transmitted through the subarachnoid space surrounding the optic nerve within the nerve sheath, especially the retrobulbar segment, unless circulation of cerebrospinal fluid is not blocked [11]. ONSD on initial brain CT may be correlated with neurologic outcomes after cardiac arrest [11, 24, 25]. Simultaneous measurement of ONSD on initial CT and intracranial pressure were correlated, and ONSD was indicative of intracranial hypertension in patients with severe traumatic brain injury. ONSD may be a good predictive marker associated with intracranial pressure [25]. Although ONSD is easy to measure on a brain CT scan, it may sometimes be difficult to measure ONSD precisely because of an inappropriate thickness of a CT section or artifacts on brain CT. In addition, similar to GWR, the cut-off values of ONSD differ between studies [11, 24, 25]. We developed a new predictive model for neurological outcomes to overcome a weakness caused by the variation in cut-off values of GWR and ONSD, and a composite of GWR, LOB/SE, and ONSD was a powerful predictor of neurological outcomes compared with each parameter alone. In real-world practice, combined CT parameters could provide improved information on early neurological prognosis in patients undergoing ECPR.

This study had several limitations. First, it was a retrospective review of medical records. Thus, the CPC scale was also retrospectively determined based on medical records. Second, we used a small heterogeneous group of subjects over a large period of time from a single institution. Over this time frame there has also been a significant change in post-arrest management which may affect patients’ outcomes over the study period. Third, the nonrandomized nature of the registry data could have resulted in selection bias. Although brain CT scans were performed within 48 h following ECPR, a major limitation of the study might be that the CT scans are performed in different time settings. However, in this study, AUC of a composite of three markers (GWR-BG + ONSD + LOB/SE) on brain CT within 15.2 h (median time) was not significantly different from that after 15.2 h (0.886 (95% CI, 0.749–0.963) vs. 0.943 (95% CI, 0.825–0.991); *p* = 0.448). One-third of patients received targeted temperature management. Hypothermia can cause coagulopathy, so therapeutic hypothermia was not attempted if patients had substantial bleeding after ECMO insertion. Therefore, surface cooling devices such as Arctic Sun were used on a limited number of patients in our study. Lastly, our study was conducted at a single institution and involved a small number of subjects. Thus, future studies with larger cohorts are needed to confirm these findings.

## Conclusions

GWR, ONSD, and LOB/SE on initial brain CT scans are associated with neurological outcome in patients who underwent ECPR. The new risk prediction model, which uses a composite of GWR, ONCD, and LOB/SE, could provide better information on neurologic outcomes in patients who underwent ECPR.

## Key messages

- Brain CT may be more helpful for predicting neurological outcome in ECPR patients under sedation because some clinical signs and examinations may also be confounded by sedation.
- GWR, ONSD, and LOB/SE on initial brain CT scans are associated with neurological outcome in patients who underwent ECPR.
- A composite of GWR, LOB/SE, and ONSD was a powerful predictor of neurological outcomes compared with each parameter alone. In real-world practice, combined CT parameters could provide improved information on early neurological prognosis in patients who underwent ECPR.

## Additional file

Additional file 1: Table S1. Specificity and sensitivity of CT markers for poor neurological outcomes. (DOCX 21 kb)

## Abbreviations

AUC: Area under the curve
BG: Basal ganglia
CC: Corpus callosum
CI: Confidence interval
CN: Caudate nucleus
CPC: Cerebral Performance Categories
CPR: Cardiopulmonary resuscitation
CT: Computed tomography
ECMO: Extracorporeal membrane oxygenation
ECPR: Extracorporeal cardiopulmonary resuscitation
GWR: Gray-to-white matter ratio
GWR-AV: Average of gray-to-white matter ratios
GWR-BG: Gray-to-white matter ratio in the basal ganglia
GWR-CO: Gray-to-white matter ratio in the cortex
GWR-SI: Simplified gray-to-white matter ratio
ICU: Intensive care unit
IQR: Interquartile range
LOB: Loss of the boundary between gray matter and white matter
MC: Medial cortex
MWM: Medial white matter
ONSD: Optic nerve sheath diameter
PIC: Posterior limb of the internal capsule
PU: Putamen
ROC: Receiver operating characteristic
ROI: Regions of interest
ROSC: Return of spontaneous circulation
SE: Cortical sulcal effacement

## References

[1] Nielsen N, Wetterslev J, Cronberg T, Erlinge D, Gasche Y, Hassager C (2013). Targeted temperature management at 33 degrees C versus 36 degrees C after cardiac arrest. N Engl J Med.

[2] Young GB (2009). Clinical practice. Neurologic prognosis after cardiac arrest. N Engl J Med.

[3] Sandroni C, Cariou A, Cavallaro F, Cronberg T, Friberg H, Hoedemaekers C (2014). Prognostication in comatose survivors of cardiac arrest: an advisory statement from the European Resuscitation Council and the European Society of Intensive Care Medicine. Intensive Care Med.

[4] Langkjaer S, Hassager C, Kjaergaard J, Salam I, Thomsen JH, Lippert FK (2015). Prognostic value of reduced discrimination and oedema on cerebral computed tomography in a daily clinical cohort of out-of-hospital cardiac arrest patients. Resuscitation.

[5] Lee YH, Oh YT, Ahn HC, Kim HS, Han SJ, Lee JJ (2016). The prognostic value of the grey-to-white matter ratio in cardiac arrest patients treated with extracorporeal membrane oxygenation. Resuscitation.

[6] Hirsch KG, Mlynash M, Jansen S, Persoon S, Eyngorn I, Krasnokutsky MV (2015). Prognostic value of a qualitative brain MRI scoring system after cardiac arrest. J Neuroimaging.

[7] Naples R, Ellison E, Brady WJ (2009). Cranial computed tomography in the resuscitated patient with cardiac arrest. Am J Emerg Med.

[8] Inamasu J, Miyatake S, Suzuki M, Nakatsukasa M, Tomioka H, Honda M (2010). Early CT signs in out-of-hospital cardiac arrest survivors: temporal profile and prognostic significance. Resuscitation.

[9] Metter RB, Rittenberger JC, Guyette FX, Callaway CW (2011). Association between a quantitative CT scan measure of brain edema and outcome after cardiac arrest. Resuscitation.

[10] Kim SH, Choi SP, Park KN, Youn CS, Oh SH, Choi SM (2013). Early brain computed tomography findings are associated with outcome in patients treated with therapeutic hypothermia after out-of-hospital cardiac arrest. Scand J Trauma Resusc Emerg Med.

[11] Hwan Kim Y, Ho Lee J, Kun Hong C, Won Cho K, Hoon Yeo J, Ju Kang M (2014). Feasibility of optic nerve sheath diameter measured on initial brain computed tomography as an early neurologic outcome predictor after cardiac arrest. Acad Emerg Med.

[12] Morimura N, Sakamoto T, Nagao K, Asai Y, Yokota H, Tahara Y (2011). Extracorporeal cardiopulmonary resuscitation for out-of-hospital cardiac arrest: a review of the Japanese literature. Resuscitation.

[13] Sakamoto T, Morimura N, Nagao K, Asai Y, Yokota H, Nara S (2014). Extracorporeal cardiopulmonary resuscitation versus conventional cardiopulmonary resuscitation in adults with out-of-hospital cardiac arrest: a prospective observational study. Resuscitation.

[14] Chen Y-S, Lin J-W, Yu H-Y, Ko W-J, Jerng J-S, Chang W-T (2008). Cardiopulmonary resuscitation with assisted extracorporeal life-support versus conventional cardiopulmonary resuscitation in adults with in-hospital cardiac arrest: an observational study and propensity analysis. Lancet.

[15] Massetti M, Tasle M, Le Page O, Deredec R, Babatasi G, Buklas D (2005). Back from irreversibility: extracorporeal life support for prolonged cardiac arrest. Ann Thorac Surg.

[16] Megarbane B, Leprince P, Deye N, Resiere D, Guerrier G, Rettab S (2007). Emergency feasibility in medical intensive care unit of extracorporeal life support for refractory cardiac arrest. Intensive Care Med.

[17] Nagao K, Kikushima K, Watanabe K, Tachibana E, Tominaga Y, Tada K (2010). Early induction of hypothermia during cardiac arrest improves neurological outcomes in patients with out-of-hospital cardiac arrest who undergo emergency cardiopulmonary bypass and percutaneous coronary intervention. Circ J.

[18] Ryu JA, Cho YH, Sung K, Choi SH, Yang JH, Choi JH (2015). Predictors of neurological outcomes after successful extracorporeal cardiopulmonary resuscitation. BMC Anesthesiol.

[19] Park SB, Yang JH, Park TK, Cho YH, Sung K, Chung CR (2014). Developing a risk prediction model for survival to discharge in cardiac arrest patients who undergo extracorporeal membrane oxygenation. Int J Cardiol.

[20] Maekawa K, Tanno K, Hase M, Mori K, Asai Y (2013). Extracorporeal cardiopulmonary resuscitation for patients with out-of-hospital cardiac arrest of cardiac origin: a propensity-matched study and predictor analysis. Crit Care Med.

[21] Cummins RO, Chamberlain DA, Abramson NS, Allen M, Baskett PJ, Becker L (1991). Recommended guidelines for uniform reporting of data from out-of-hospital cardiac arrest: the Utstein Style. A statement for health professionals from a task force of the American Heart Association, the European Resuscitation Council, the Heart and Stroke Foundation of Canada, and the Australian Resuscitation Council. Circulation.

[22] Kang MJ, Lee TR, Shin TG, Sim MS, Jo IJ, Song KJ (2014). Survival and neurologic outcomes of out-of-hospital cardiac arrest patients who were transferred after return of spontaneous circulation for integrated post-cardiac arrest syndrome care: another feasibility of the cardiac arrest center. J Korean Med Sci.

[23] Torbey MT, Selim M, Knorr J, Bigelow C, Recht L (2000). Quantitative analysis of the loss of distinction between gray and white matter in comatose patients after cardiac arrest. Stroke.

[24] Legrand A, Jeanjean P, Delanghe F, Peltier J, Lecat B, Dupont H (2013). Estimation of optic nerve sheath diameter on an initial brain computed tomography scan can contribute prognostic information in traumatic brain injury patients. Crit Care.

[25] Sekhon MS, Griesdale DE, Robba C, McGlashan N, Needham E, Walland K (2014). Optic nerve sheath diameter on computed tomography is correlated with simultaneously measured intracranial pressure in patients with severe traumatic brain injury. Intensive Care Med.

[26] Cummins RO, Chamberlain D, Hazinski MF, Nadkarni V, Kloeck W, Kramer E (1997). Recommended guidelines for reviewing, reporting, and conducting research on in-hospital resuscitation: the in-hospital 'Utstein style'. Circulation.

[27] DeLong ER, DeLong DM, Clarke-Pearson DL (1988). Comparing the areas under two or more correlated receiver operating characteristic curves: a nonparametric approach. Biometrics.

[28] Schisterman EF, Perkins NJ, Liu A, Bondell H (2005). Optimal cut-point and its corresponding Youden Index to discriminate individuals using pooled blood samples. Epidemiology.

[29] Ruopp MD, Perkins NJ, Whitcomb BW, Schisterman EF (2008). Youden Index and optimal cut-point estimated from observations affected by a lower limit of detection. Biomed J.

[30] Neumar RW, Shuster M, Callaway CW, Gent LM, Atkins DL, Bhanji F (2015). Part 1: Executive summary: 2015 American Heart Association guidelines update for cardiopulmonary resuscitation and emergency cardiovascular care. Circulation.

[31] Shin TG, Choi JH, Jo IJ, Sim MS, Song HG, Jeong YK (2011). Extracorporeal cardiopulmonary resuscitation in patients with inhospital cardiac arrest: a comparison with conventional cardiopulmonary resuscitation. Crit Care Med.

[32] Samaniego EA, Mlynash M, Caulfield AF, Eyngorn I, Wijman CA (2011). Sedation confounds outcome prediction in cardiac arrest survivors treated with hypothermia. Neurocrit Care.

