# Supplementary material for: The association of findings on brain computed tomography with neurologic outcomes following extracorporeal cardiopulmonary resuscitation
Source: Crit Care. 2017 Jan 25;21:15. doi: 10.1186/s13054-017-1604-6 (PMC5264281; doi:10.1186/s13054-017-1604-6)
Supplement: Additional file 1: Table S1. — Specificity and sensitivity of CT markers for poor neurological outcomes. (DOCX 21 kb) [file 13054_2017_1604_MOESM1_ESM.docx]

**Supplementary Table1** Specificity and sensitivity of CT markers for poor neurological outcomes

|  | Cut-off value | Specificity (%) | Sensitivity (%) | PPV (%) | NPV (%) | AUC (95% CI) | *p* value |
| --- | --- | --- | --- | --- | --- | --- | --- |
| Gray-to-white matter Ratio (GWR)  GWR-BG (basal ganglia)  GWR-CO (cortical)  GWR-SI (simplified)  GWR-AV (average) | 1.14  1.23  1.11  1.13  1.02  1.25  1.14  1.23 | 100  84.2  100  94.7  100  73.7  100  79.0 | 34.8  60.9  34.8  43.5  21.7  65.2  39.1  60.9 | 100  82.4  100  90.9  100  75.0  100  77.8 | 55.9  64.0  55.9  58.1  51.4  63.6  57.6  62.5 | 0.792 (0.639 – 0.901)  0.650 (0.487 – 0.790)  0.706 (0.545 – 0.836)  0.746 (0.588 – 0.867) | 0.001  0.098  0.023  0.007 |
| Optic nerve sheath diameter (mm) | 6.69  5.86 | 100  73.7 | 21.7  60.9 | 100  73.7 | 51.4  60.9 | 0.745 (0.587 – 0.867) | 0.007 |
| Loss of boundary (LOB) |  |  |  |  |  | 0.773 (0.628 – 0.919) | < 0.001 |
| Cortical sulcal effacement (SE) |  |  |  |  |  | 0.773 (0.628 – 0.919) | < 0.001 |

CT, computed tomography; PPV, positive predictive value; NPV, negative predictive value; AUC, area under the curve; CI, confidence interval.
